# Women’s Childbirth Experiences During COVID-19 Pandemic in Indonesia

**DOI:** 10.1590/0034-7167-2022-0640

**Published:** 2023-10-09

**Authors:** Belet Lydia Ingrit, Joice Cathryne, Shinta Marina J.P Sihaloho, Angelika Quisessa

**Affiliations:** I Universitas Pelita Harapan. Lippo Karawaci, Tangerang, Indonesia; II Minerva Clinic. Palangkaraya, Central Kalimantan, Indonesia

**Keywords:** COVID-19, Childbirth, Experience, Indonesia, Pandemic, COVID-19, Parto, Morte, Indonésia, Pandemias, COVID-19, Parto, Experiencia, Indonesia, Pandemia

## Abstract

**Objective:**

Explore in depth the experiences of women giving birth during the COVID-19 pandemic in Indonesia.

**Methods:**

Qualitative research with a descriptive phenomenological. A total of 16 participants did not suffer from COVID-19 and gave birth during the COVID-19 pandemic in the community setting area.

**Results:**

There are five themes: 1: Feelings of anxiety and fear of giving birth in hospitals are experienced by women giving birth during the COVID-19 pandemic, 2. Screening for COVID and health protocols applied in hospitals during the COVID-19 pandemic, 3. Support from husbands, families, and health workers is needed when giving birth during the COVID-19 pandemic, 4. Lack of family visits hours is an obstacle during childbirth, particularly during the COVID-19 pandemic, and 5: Bonding attachment between mother and baby while at the hospital.

**Conclusion:**

Optimal maternity nursing care and supporting health facilities and public policies will help mothers give birth during the COVID-19 pandemic in Indonesia.

## RESUMO

**Objetivo:**

Explorar em profundidade as experiências de mulheres que deram à luz durante a pandemia de COVID-19 na Indonésia.

**Métodos:**

Pesquisa qualitativa com abordagem fenomenológica descritiva. Um total de 16 participantes que não sofriam de COVID-19 deram à luz durante a pandemia de COVID-19 no ambiente comunitário.

**Resultados:**

Há cinco temas: 1: Sentimentos de ansiedade e medo de dar à luz em hospitais são vivenciados por mulheres que dão à luz durante a pandemia de COVID-19, 2. Triagem para COVID e protocolos de saúde aplicados em hospitais durante a pandemia de COVID-19, 3. O apoio de maridos, famílias e profissionais de saúde é necessário durante o parto na pandemia de COVID-19, 4. A falta de horário para visitas familiares é um obstáculo durante o parto, principalmente durante a pandemia de COVID-19, e 5: Vínculo entre mãe e bebê no hospital.

**Conclusão:**

Os cuidados ideais de enfermagem na maternidade e o apoio das unidades de saúde e políticas públicas ajudarão as mães a dar à luz durante a pandemia de COVID-19 na Indonésia.

## RESUMEN

**Objetivo:**

Explorar en profundidad las experiencias de las mujeres que dieron a luz durante la pandemia de COVID-19 en Indonesia.

**Métodos:**

Investigación cualitativa con enfoque fenomenológico descriptivo. Un total de 16 participantes que no sufrieron de COVID-19 dieron a luz durante la pandemia de COVID-19 en el entorno comunitario.

**Resultados:**

Hay cinco temas: 1: Los sentimientos de ansiedad y miedo de dar a luz en los hospitales son experimentados por las mujeres que dan a luz durante la pandemia de COVID-19, 2. Detección de COVID y protocolos de salud aplicados en los hospitales durante la pandemia de COVID-19, 3. Se necesita el apoyo de esposos, familias y trabajadores de la salud cuando se da a luz durante la pandemia de COVID-19, 4. La falta de horarios de visitas familiares es un obstáculo durante el parto, particularmente durante la pandemia de COVID-19, y 5: Vínculo entre madre y bebé mientras están en el hospital.

**Conclusión:**

la atención de enfermería de maternidad óptima y el apoyo de los centros de salud y las políticas públicas ayudarán a las madres a dar a luz durante la pandemia de COVID-19 en Indonesia.

## INTRODUCTION

The COVID-19 pandemic has become a worldwide health problem and has led to health workers experiencing fatigue in overcoming existing health problems^(1)^. COVID-19 first appeared in Wuhan, China at the end of December 2019^(2)^ and this virus has been in Indonesia since March 2, 2020, with two patients identified as positive for COVID-19^(3)^. This virus can spread between humans through respiratory droplets and direct contact^(4)^. COVID-19 has become a global pandemic including in Indonesia, which causes anxiety for the community because the community is required to adopt from normal life to an anxious life due to the pandemic.

Childbirth is an important event in life and becomes a personal experience for women^(5)^. This experience influences the long-term health and well-being of women^(6)^. Nursing care during childbirth is to provide physical well-being and emotions, which includes changes throughout pregnancy, childbirth, and recovery, as also the transition to parenthood that will be experienced in giving birth^(7)^. The experience of giving birth makes a great impression on a woman. Satisfaction with giving birth will increase women’s adaptation to their role as a mother^(8)^.

During childbirth, women usually have fears related to the process, the effects of childbirth, concern about the health of the mother and the baby, and fears about treatment from health workers^(8)^. A previous study stated that women who experienced fears and anxiety before the process of giving birth required a professional helper because, if not, the mother will worry and in the future, this will cause bonding problems between the mother and their child^(9)^.

Fear and anxiety from mothers who gave birth during the COVID-19 pandemic e will naturally increase. Mothers will face the possibility of giving birth in a hospital and delivery plans that have been made in advance, such as where to labor and who will accompany childbirth, is a question during a pandemic^(10)^. Afraid and anxiety if not handled properly will certainly hamper the labor process as a result of the inhibition of the effects of catecholamines associated with the stress response on uterine contractions^(8)^.

Although research in various fields regarding the COVID-19 disease is overwhelming, until now qualitative research during the COVID-19 pandemic on childbirth or experience after giving birth is still limited. It becomes very important because maternal health is one of the implementations of 2030 for Sustainable Development^(11)^. As the importance of women’s experiences during childbirth will influence the bonding attachment between mother and baby and the role of women as a mother, the researcher wants to dig deeper into the experience of giving birth during the COVID-19 pandemic.

## OBJECTIVE

To explore more in-depth women’s experiences during childbirth in the COVID-19 pandemic situation to develop nursing services, especially maternity nursing.

## METHODS

### Ethical aspects

This study was approved by the Mochtar Riady Institute for Nanotechnology Ethics Committee with Protocol Number: 2005010-04.

### Study design, location, and period

This is a qualitative study with a descriptive phenomenology design, in the period June-September 2020. Participants in this study were women who did not suffer from COVID-19 disease and who gave birth during the pandemic. Data collection took place via virtual meetings via Zoom and meetings directly on-site with in-depth interviews.

### Population or sample, inclusion and exclusion criteria

Participants in this study were women who had a history of birth during the COVID-19 pandemic in Indonesia. This study includes 16 participants with inclusion criteria: women who gave birth during the COVID-19 pandemic, women with no COVID-19, and participants willing to be research participants.

Women with COVID-19 disease were unable to conduct the interviews during the study period due to interference, physical and psychological, and participants with disorders such as postpartum hemorrhage and psychological disorders such as baby blues syndrome and birth trauma were excluded from this study.

### Study protocol

The calculation of the sample size was performed using the purposive sampling method and determined by the number of participants of women with birth history during the pandemic. The participants were selected until the data reached the saturation point (method of saturation) and no more new topics were generated.

Researchers will establish a relationship with participants and communicate the purpose and benefits of the study as well as information consent (informed consent). In this study, participants have the right to decide whether to accept this research or not. When participants agreed, they filled out the informed consent form. The participant data will be kept confidential and will be used only for research purposes and when all the data and records are no longer available to be used, they will be destroyed immediately. During interviews or data collection, the researcher maintains the participant’s comfort at the place or location that has been decided by the participant and the time contract decided by the participant (protection from discomfort).

**Table 1:** Description of participants’ characteristics

Code	Age (years)	Location	Education	Ethic	Job-status	Obstetric status
P1	25	Tangerang	Senior High School	Dayak	Housewife	P1A0
P2	30	Jakarta	Bachelor	Batak	Employee	P1A0
P3	30	Tangerang	Magister	Batak	Lecturer	P1A0
P4	31	Tangerang	Bachelor	Batak	Employee	P2A0
P5	30	Jakarta	Bachelor	Batak	Employee	P1A0
P6	30	Jakarta	Bachelor	Batak	Nurse	P2A0
P7	29	Surabaya	Bachelor	Minahasa	Housewife	P1A0
P8	36	Tangerang	Diploma	Batak	Housewife	P1A0
P9	31	Manado	Magister	Manado	Housewife	P2A0
P10	36	Medan	Diploma	Batak	Midwife	P3A0
P11	24	Maluku	Senior High School	Ambon	Housewife	P1A0
P12	32	Palangkaraya	Bachelor	Dayak	Pastor	P2A0
P13	28	Flores	Senior High School	Danga	Housewife	P3A0
P14	26	Papua	Bachelor	Maybe at	Housewife	P1A0
P15	27	Palu	Diploma	Poso	Nurse	P2A0
P16	33	Tangerang	Magister	Batak	Lecturer	P1A0

The researcher arranged the interview schedule directly and indirectly by using an in-depth interview method using field notes with the time and place specified agreed upon. Interviews were conducted with an interview questions guide. The process was recorded with a tape recorder or, if using virtual meetings, the meeting was recorded. The time for an interview is 60-90 minutes. During the interview, researchers paid attention to non-verbal secondary data such as intonation, silence, laughter, participants’ gestures, and facial expressions.

The questions in this study are adjusted with the research objective, they are: (1) How is the experience of the mother during childbirth in the COVID-19 pandemic?, (2) How are you feeling now?, (3) How are your obstacles during childbirth in the COVID-19 pandemic?, (4) How do family and health workers support during childbirth in the COVID-19 pandemic?, (5) Do you have any other experiences that would you like to share?

After collecting the data from the interview, then the next step is to process the data. The process of data analysis used the thematic content analysis stages of Collaizi, which involves eight steps.

## RESULTS

The 16 women participants are from Jakarta, Tangerang, Surabaya, Manado, Medan, Maluku, Palangkaraya, Flores, Palu, and Papua. The average age is between 25-36 years old with an education background from Senior High School until Magister in Nursing. Most of them work as housewives and working as a nurse. lecturer and employee. Obstetric status from participants mostly had one or two children and never had an abortus history. Their ethnic groups are Dayak, Bataknese, Minahasa, Poso, and Manado.

The results of this study are the results of interviews analysis conducted on 16 participants and field notes taken during in-depth interviews. Themes are 1: Feelings of anxiety and fear of giving birth in hospitals are experienced by women giving birth during the COVID-19 pandemic, 2. Screening for COVID and health protocols applied in hospitals during the COVID-19 pandemic, 3. Support from husbands, families, and health workers is needed when giving birth during the COVID-19 pandemic, 4. Lack of family visits hours is an obstacle during childbirth, particularly during the COVID-19 pandemic, and 5: Bonding attachment between mother and baby while at the hospital.

## DISCUSSION

Theme 1 is feelings of anxiety and fear of giving birth in the hospital experienced by women giving birth during the COVID-19 pandemic. Following are some expressions from participants:

*The experience, yes, it’s mixed, one of them, yes, there must be a sense of worry too.* (P1)

*The experience, first of all, is, what is fear. So what is it called, more worried.* (P4)

*My experience when giving birth during this pandemic was feeling of anxiety, I was constantly worried because pregnant women have a very high risk to get COVID-19.* (P6)

Feelings of anxiety and fear of giving birth during the COVID-19 pandemic are supported by Pane’s research in 2021 which said that most pregnant women in the third-trimester experience mild to moderate anxiety caused by the mother’s fear of being infected and infected with COVID-19^(12)^. Angesti & Febriyana (2021) said that there is relationship between the anxiety level and knowledge of pregnant women in third trimester with readiness for childbirth during the COVID-19 Pandemic^(13)^. In another study, 44.3% of mothers with gestational age third trimester, 38.6% of mothers with gestational age in the second trimester, and 17.1% of mothers with first gestational age. Mothers with third-semester pregnancies have higher levels of increased anxiety and fear associated with the birth process^(14)^.

Anxiety and stress are also experienced by adolescent mothers during the birth process during the COVID-19 pandemic and getting poor quality care during the COVID-19 pandemic. Anxiety was experienced for 3-4 months after giving birth during the COVID-19 pandemic^(15)^. Other research said that anxiety experienced by respondents was caused by the mother’s fear of being infected and infecting COVID-19. Results from Pane’s (2021) research showed that 60.6% had mild to moderate anxiety and 33.3% experienced severe anxiety during the pandemic^(12)^.

The feeling of fear of giving birth in the hospital was also expressed by eight of 16 participants. Here are some expressions from participants:

*The experience is that going to the hospital is also feeling of fear and doubt.* (P5)

*Even though we are afraid to go to hospitals now because of Corona.* (P7)

*I wanted to give birth at the hospital at first, but because of COVID, the hospital was closed so I gave birth at the Public Health Center.* (P14)

Research by Suwarti, Ratnasari & Winarti (2021) said that there are changes in psychological, physical and social aspects, based on the pregnant women experience during the COVID-19 pandemic^(16)^. Research conducted by Loret de Mola et al. in Brazil found increasing cases or increasing prevalence of mental health of pregnant women. There is a high rate of depression and anxiety in pregnant women during the COVID-19 pandemic^(17)^. Not only the COVID-19 pandemic factor, but other factors will also affect maternal anxiety, for example, being less stable emotionally, and having bad experiences in previous deliveries and educational status will affect the mother’s fear of giving birth and also childbirth experience^(18)^.

The second theme is screening for COVID-19 and health protocols applied in hospitals during the COVID-19 pandemic. This is stated by several participants:

*I spend more time on the COVID-19 test.* (P1)

*But, because of this pandemic, we entered Emergency Room and had to do all rapid tests, blood tests, and lung tests.* (P5)

*But if there are several hospitals here to be a referral for COVID-19, so, for example, rapid positive, sent to the COVID-19 referral hospital. After that is swab test.* (P15)

The pattern applied by the hospital in accepting pregnant women during the pandemic through COVID-19 screening and health protocols is in line with Padilla et al.’s research which said that health protocols applied for breastfeeding mothers and pregnant women caregivers understand the correct use of masks and cough etiquette, maintain personal hygiene in the home environment and when visiting healthcare facilities^(15)^.

Participants in Tangerang also experienced giving birth. Different procedure pregnancy checks are carried out by pregnant women before and during the COVID-19 pandemic. This theme was made by Suwarti et al.’s research which said that health protocol for pregnant women is carried out during the COVID-19 pandemic by applicable regulations written in the Guidelines for Pregnant Women, Postpartum Mothers, and Newborns during Social Distancing^(16)^.

Research in Singapore said that age, ethnicity, and occupation will affect attitudes and precautions for pregnant women in implementing health protocols during pregnancy during the COVID-19 pandemic^(19)^. COVID-19 also affected the number of births through the SC process. SC procedure is carried out on mothers who give birth with a positive case of COVID-19^(20)^. This procedure is for safety and health protocols compared to the risk of having a vaginal delivery. The impact of COVID-19 pandemic will affect maternal care. Nursing care for a mother in preparation for labor should be defined as timing, mode of delivery, safe delivery, room preparation, anesthetic choice, and newborn observation during the pandemic^(21)^.

The third theme of this study is support from husbands, families, and health workers is needed when giving birth during the COVID-19 pandemic. This is stated by the participants:

*As for family support, it’s very supportive, everyone advises to be calmer, don’t panic too much, especially from my husband.* (P2)

*I am grateful that the medical personnel did strengthen me, I am grateful because they are indeed supportive from their knowledge, they are also people who fear the Lord.* (P12)

*From my husband, I have been very supportive and also from my family. I was stressed because I must give birth during a pandemic, but my husband always support me.* (P15)

Research in United Kingdom said that COVID-19 pandemic effects on prenatal mental health, antenatal attachment dan social support^(22)^. They found that pandemic has affected UK expectant mother’s mental health by increasing prevalence of depression (47%), anxiety (60) and stress related by psychological impact of COVID-19 (40%). This study also said that higher social support acted as a protective factor and associated with lower anxiety. The pregnant women were under intense stress during the COVID-19 outbreak. Mortazavi & Ghardasi (2021) said that the health system is necessary for alleviating pregnant women’s difficulties in situations like the COVID19 epidemic. Virtual training classes and virtual counseling may enhance the peace and tranquility of pregnant women^(23)^.

The fourth theme in this study is lack of family visits hours is an obstacle during childbirth, particularly during the COVID-19 pandemic. This is stated by several participants:

*Well, I can’t wait there, I can’t wait and cannot visit*. (P3)

*So no one visits at all, it’s just the two of you and your husband.* (P6)

*The obstacle is that you can’t be accompanied by anyone other than your husband, the relatives are same not allowed to visit.* (P12)

The Ministry of Health has published guidelines for the management of pregnancy, childbirth, and postpartum as well as new babies born during the COVID-19 pandemic^(24)^. The Ministry of Health said that a limited number of health workers can enter the treatment room and only one person can accompany mothers who are giving birth by using appropriate PPE (Personal Protective Equipment). Not only services in hospitals, antenatal, and intranatal in healthcare community facilities also follow the rules or policies of the government. A family or husband can accompany during the pandemic with the condition that the family or husband has a negative swab test. Nurses and health workers can provide regular health education and information about COVID-19 either through social media or electronic media as well as postpartum visits through home visits and online monitoring^(14)^.

The fifth theme is the existence of bonding attachment between mother and baby while in the hospital. This was stated by several participants:

*Well take care together just usually after six hours of observation.* (P5)

*Stay hospitalized in six hours after observation from birth until going to baby room with their parents.* (P6)

Bonding attachment is very necessary for mothers giving birth during the COVID-19 pandemic. Research conducted in Medan by Siregar et al. stated that the existence of a social distancing policy will cause anxiety for mothers during the childbirth hospital treatment process^(25)^. Bonding attachments can also be formed during the Early Initiation of Breastfeeding^(26)^. But in this study, Early Initiation Breastfeeding was difficult because of the pandemic condition. Support for breastfeeding mothers during pandemics is also needed so the process of exclusive breastfeeding can run well. Research by Galego et al. in Spain said that support groups of breastfeeding mothers (Facebook or Instagram) have been shown to provide support and sources of information during the pandemic^(27)^.

Mayopoulos’s research said that postpartum mothers with COVID-19 had a separate room and this can cause psychological problems when going through the postpartum care process compared to postpartum mothers who received assistance from their husbands and were not separated from their babies^(28)^.

### Study limitations

This study is limited by the place because of the pandemic situation, not all participants can meet face-to-face directly when interviewed.

### Contributions to nursing, health, and public policies

This study can contribute to directing interprofessional healthcare like maternity nursing in the hospice community. Knowing the experience of women when childbearing, and postpartum care will enable maternity nurses to find the best nursing care in collaboration with other health workers in hospitals and community settings.

## FINAL CONSIDERATIONS

A good experience postpartum and not causing trauma will greatly affect the psychological condition of the mother. Optimal maternity nursing care and supporting health facilities will help mothers give birth during the COVID-19 pandemic in Indonesia.
